# Supplementary material for: Ultra-high field strength electroporation enables efficient DNA transformation and genome editing in nontuberculous mycobacteria
Source: Microbiol Spectr. 2025 Sep 8;13(10):e01944-25. doi: 10.1128/spectrum.01944-25 (PMC12502714; doi:10.1128/spectrum.01944-25)
Supplement: Supplemental figures and tables — Fig. S1 and S2, and Tables S1 and S2. [file spectrum.01944-25-s0001.pdf]

# Ultra-high field strength electroporation enables efficient DNA transformation and genome editing in nontuberculous mycobacteria

Daoyan Tang[a,#], Minggui Wang[b,#], Dan Wang[b], Danni Yang[b], Yi Cai[d], Tao Luo[e], Jianqing He[a,c], Qinglan Wang[b,c]

a. Department of Respiratory and Critical Care Medicine, West China Hospital, Sichuan University, Chengdu, China.

b. Institute of Respiratory Health, Frontiers Science Center for Disease-related Molecular Network, West China Hospital, Sichuan University, Chengdu, China.

c. State Key Laboratory of Respiratory Health and Multimorbidity, West China Hospital, Sichuan University, Chengdu, China.

d. Guangdong Provincial Key Laboratory of Infection Immunity and Inflammation, Department of Pathogen Biology, Shenzhen University Medical School, Shenzhen, China.

e. Department of Pathogen Biology, West China School of Basic Medical Sciences & Forensic Medicine, Sichuan University, Chengdu, China.

**Fig. S1**

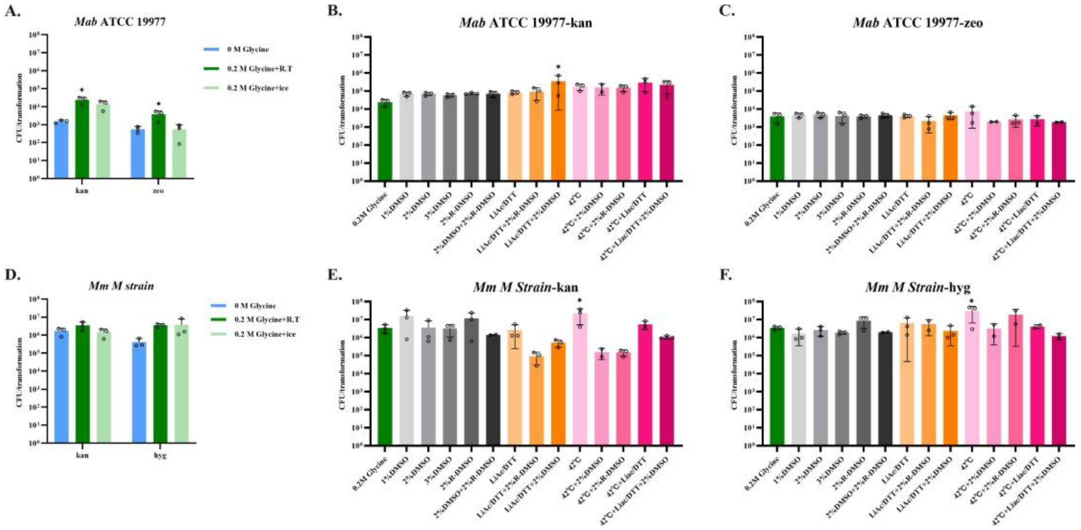


**Figure. S1 | Enhanced Electrotransformation Efficiency in Nontuberculous Mycobacteria through Cell Wall–Weakening and Heat Shock Pretreatments.**

(A) Effect of glycine pretreatment and competent cell preparation temperature (ice vs R.T) on transformation efficiency in *M. abscessus*. Transformants were selected on media containing kanamycin (Kan) or zeocin (Zeo). R.T: room temperature, ~25°C. Ice: Cultures were placed on ice for 1 h prior to centrifugation, with all subsequent steps also performed at 4 °C (centrifugation) or on ice (cell handling) during competent cell preparation. (B-C) Impact of individual or combined treatments—dimethyl sulfoxide (DMSO), lithium acetate with dithiothreitol (LiAc/DTT), and 42 °C heat shock—on transformation efficiency in *M. abscessus* under Kan (B) or Zeo (C) selection. 2%R-DMSO (2% DMSO in recovery medium). (D) Effect of glycine pretreatment and preparation temperature of competent cells on electrotransformation efficiency in *M. marinum* under Kan or hygromycin (Hyg) selection. (E-F) Effect of DMSO, LiAc/DTT, 42 °C heat shock, or their combinations on transformation efficiency in *M. marinum* under Kan (E) or Hyg (F) selection. All experiments were independently repeated 3 times. *P*-value: *, <0.05; Dunnett's 1-way ANOVA.

**Fig. S2**

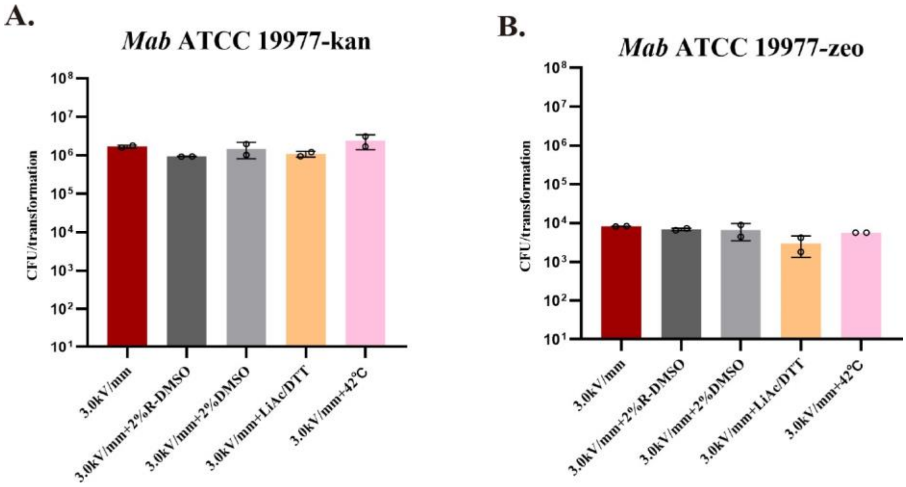


Fig. S2. Synergistic effects of ultra-high electric field strength with DMSO, LiAc/DTT, or 42°C treatment on transformation efficiency in *M. abscessus* under Kan (A) or Zeo (B) selection.

**Fig. S3**

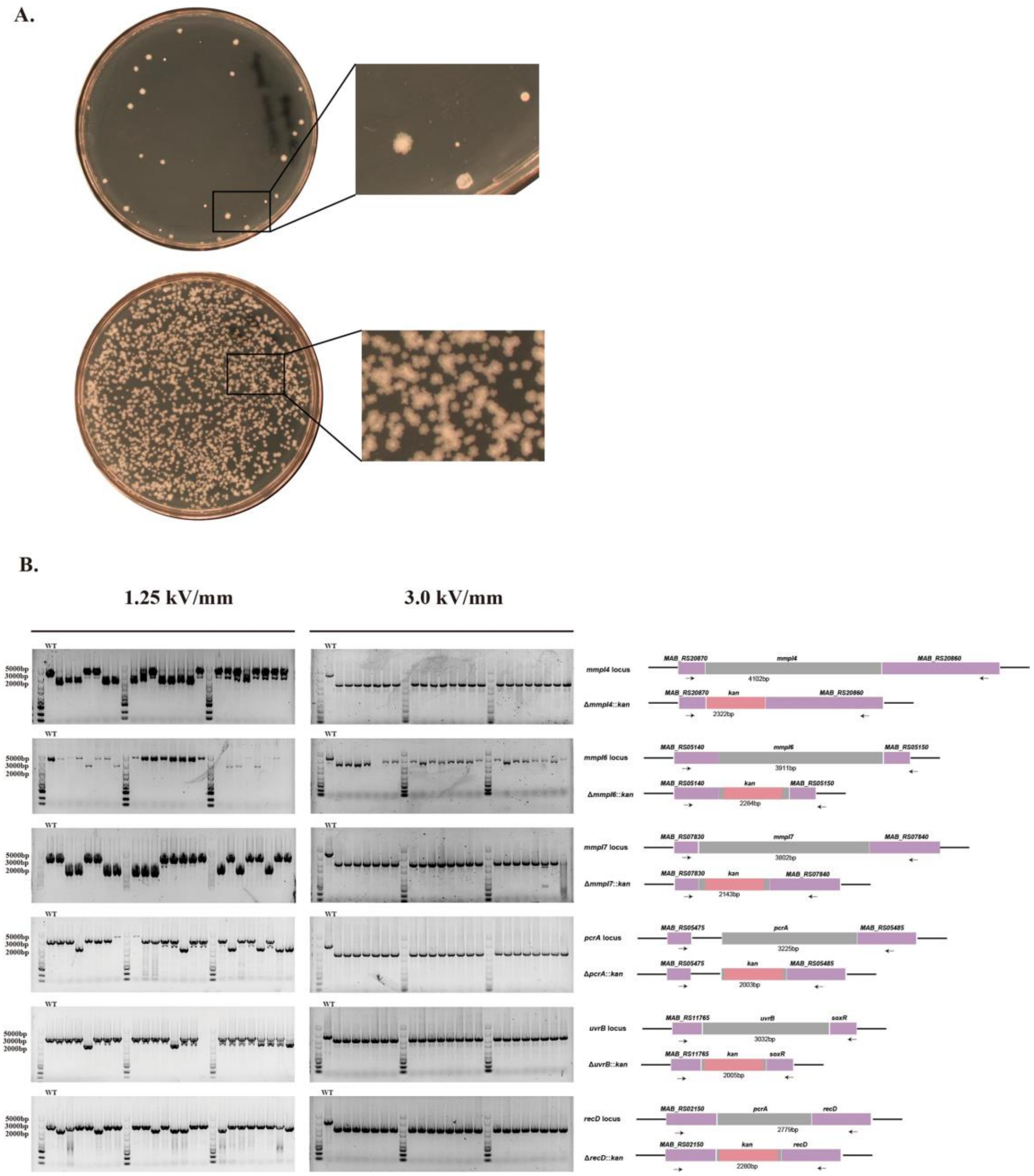


Fig. S3. Ultra-high electric field strength electroporation enhances gene knockout efficiency in *M. abscessus*.

(A) Representative images of transformant colonies following electroporation of allele exchange substrates targeting mmpl4 in *M. abscessus* expressing RecET recombinase.

Transformants were selected on kanamycin-containing plates.Top, ultra-high electric field strength electroporation; bottom, conventional field strength. Rough colony morphology indicates successful mmpl4 disruption. (B) Validation of gene knockouts by colony PCR and agarose gel electrophoresis for six target loci: mmpl4, mmplo, mmpl7, prcA, uvrB, and recD.

**Supplementary Table 1.** Bacterial strains and plasmids used in this study.

| Strain or Plasmid | Description | Source |
|---|---|---|
| *Mycobacterium abscessus* ATCC 19977™ | Wild-type strain | Sichuan University |
| *Mycolicibacterium smegmatis* mc²155 (ATCC 700084™) | Wild-type strain | Sichuan University |
| *Mycobacterium marinum* M strain | Wild-type strain | Sichuan University |
| *Mycobacterium kansasii* SC196 | Clinical isolate from West China Hospital | This study |
| *M. abscessus* ATCC 19977™ $GPL^-$ | Derivative of ATCC 19977 with deletion of *mps2*, resulting in a smooth-to-rough colony morphology shift | This study |
| *M. abscessus* ATCC 19977 (pJV53-zeo-mScarlet) | ATCC 19977 transformed with pJV53-zeo-mScarlet, expressing RecET recombinase under an acetamide-inducible promoter and the fluorescent marker mScarlet | This study |
| *M. abscessus* ATCC 19977 (pKM461-dkan-zeoR) | ATCC 19977 transformed with pKM461-dkan-zeoR, expressing RecT recombinase under a tetracycline-inducible promoter; contains a non-functional *kanR* (E118*), zeocin resistance, and mScarlet | This study |
| pQL037 | pJV53-based vector lacking che9c phage *recET* genes; encodes mScarlet for fluorescence | This study |
| pQL038 | pKM461 derivative with kanR replaced by zeoR and insertion of mScarlet | This study |
| pQL039 | pKM461 derivative with kanR replaced by hygromycin resistance cassette and mScarlet | This study |
| pJV53-zeo-mScarlet | Modified pJV53 plasmid with *kanR* replaced by *zeoR*; expresses RecET under acetamide-inducible promoter and mScarlet | This study |

| | | |
|---|---|---|
| pKM461-dkan-zeoR | pKM461 backbone with inactivated kanR (E118*), zeoR, and mScarlet; RecT expression under tetracycline-inducible promoter | This study |
| pUC19-MabmmpL4aF-Kan | pUC19 backbone, inserted *mmpl4*F1-kanR-*mmpl4*F2 | This study |
| pUC19-MabmmpL6F-Kan | pUC19 backbone, inserted *mmpl6*F1-kanR-*mmpl6*F2 | This study |
| pUC19-MabmmpL7F-Kan | pUC19 backbone, inserted *mmpl7*F1-kanR-*mmpl7*F2 | This study |
| pUC19-MabPcrAF-Kan | pUC19 backbone, inserted *prcA*F1-kanR-*prcA*F2 | This study |
| pUC19-MabrecDF-Kan | pUC19 backbone, inserted *recD*F1-kanR-*recD*F2 | This study |
| pUC19-MabuvrBF-Kan | pUC19 backbone, inserted *uvrB*F1-kanR-*uvrB*F2 | This study |

**Supplementary Table 2. Primers used for allelic exchange and PCR verification of gene deletions.**

| **Primer name** | **Sequence (5′–3′)** | **Use** |
|---|---|---|
| mmpL4aF1Fw | **GTAAAACGACGGCCAGTGAATTC**ccctccaacgtacgttttggacacgt | Forward primer for left flank of AES (*mmpl4* deletion) |
| mmpL4aF1Rv | **CCGGGGCGTCAGGCGC**gtcgtccgcgctcatgcggcct | Reverse primer for left flank of AES (*mmpl4* deletion) |
| mmpL4aF2Fw | **AAGCCCGCCATTAGGCG**tccgctcggtactgcagaggaatt | Forward primer for right flank of AES (*mmpl4* deletion) |
| mmpL4aF2Rv | **GACCATGATTACGCCAAGCTT**cgacgctgcgatatgtggtgtagtagg | Reverse primer for right flank of AES (*mmpl4* deletion) |
| mmpL6F1Fw | **GTAAAACGACGGCCAGTGAATTC**gtcgtcgatcagcgccttgcgct | Forward primer for left flank of AES (*mmpl6* deletion) |
| mmpL6F1Rv | **CCGGGGCGTCAGGCGC**agaacaagacgatggggacggcaag | Reverse primer for left flank of AES (*mmpl6* deletion) |
| mmpL6F2Fw | **AAGCCCGCCATTAGGCG**ctggttctggtggcctatcaacatacgt | Forward primer for right flank of AES (*mmpl6* deletion) |
| mmpL6F2Rv | **GACCATGATTACGCCAAGCTT**tcgcggacaagggtgtccgtttca | Reverse primer for right flank of AES (*mmpl6* deletion) |
| mmpL7F1Fw | **GTAAAACGACGGCCAGTGAATT**Ctgacgtccaagacgttcggcgt | Forward primer for left flank of AES (*mmpl7* deletion) |

| | | |
|---|---|---|
| mmpL7F1Rv | **CCGGGGCGTCAGGCGCT**ggttatgtgcggtgctttccacctgt | Reverse primer for left flank of AES (*mmpl7* deletion) |
| mmpL7F2Fw | **AAGCCCGCCATTAGGCG**aacagccggggtgctgtcctacct | Forward primer for right flank of AES (*mmpl7* deletion) |
| mmpL7F2Rv | **GACCATGATTACGCCAAGCTT**agcaggtgcaggcgaatgtggaa | Reverse primer for right flank of AES (*mmpl7* deletion) |
| pcrAF1Fw | **GTAAAACGACGGCCAGTATCGAT**ggtccggttgttctcgacccatagg | Forward primer for left flank of AES (*pcrA* deletion) |
| pcrAF1Rv | **CCGGGGCGTCAGGCGC**aacgcgtcgctggaggcact | Reverse primer for left flank of AES (*pcrA* deletion) |
| pcrAF2Fw | **AAGCCCGCCATTAGGCG**acttcgctcatcgacttcggcagt | Forward primer for right flank of AES (*pcrA* deletion) |
| pcrAF2Rv | **GACCATGATTACGCCAAGCTT**tatcgaggccaccgctgactggtt | Reverse primer for right flank of AES (*pcrA* deletion) |
| uvrBF1Fw | **GTAAAACGACGGCCAGTGAATTC**gggctggaaccgtgtccacatt | Forward primer for left flank of AES (*uvrB* deletion) |
| uvrBF1Rv | **CCGGGGCGTCAGGCGC**aacgcgtcgctggaggcact | Reverse primer for left flank of AES (*uvrB* deletion) |
| uvrBF2Fw | **AAGCCCGCCATTAGGCG**acttcgctcatcgacttcggcagt | Forward primer for right flank of AES (*uvrB* deletion) |
| uvrBF2Rv | **GACCATGATTACGCCAAGCTT**tatcgaggccaccgctgactggtt | Reverse primer for right flank of AES (*uvrB* deletion) |
| recDF1Fw | **GTAAAACGACGGCCAGTGAATTC**tatcgagcgaaccggagatcgccga | Forward primer for left flank of AES (*recD* deletion) |
| recDF1Rv | **CCGGGGCGTCAGGCGC**atgccaacgccgtcagccgct | Reverse primer for left flank of AES (*recD* deletion) |
| recDF2Fw | **AAGCCCGCCATTAGGCG**actgcgccacagcccgaactgat | Forward primer for right flank of AES (*recD* deletion) |
| recDF2Rv | **GACCATGATTACGCCTCTAGA**cgatctttagacggacggtcggtca | Reverse primer for right flank of AES (*recD* deletion) |
| oligo1 | **CATTCCAGGTATTAGAAGAATATCCTGATTCAGGTGAAAATATTGTTGATGCGCTGGCAGTGTTCCTGCG** | Oligonucleotide for introducing point mutations in *kanR* |
| oligo2 | **CGCAGGAACACTGCCAGCGCATCAACAATATTTTCACCTGAATCAGGATATTCTTCTAATACCTGGAATG** | Complementary strand for *kanR* point mutation |
| gyrAAsp96Asn | **gtaactatcacccccacggtgacgcgtccatctacAacaccctcgtgcgtatggcccagccttggtcgct** | Oligonucleotide for *gyrA* Asp96Asn point mutation |
| mab-mmpl4-seqFw | gcctcggggtcataaagagg | Forward primer for colony PCR and sequencing of *mmpL4*-replaced allele |
| mab-mmpl4-seqRv | cctgcgcggtaccttgatta | Reverse primer for colony PCR and sequencing of *mmpL4*-replaced allele |

| | | |
|---|---|---|
| mab-mmpl6-seqFw | gcgggacatgtctcaaccaa | Forward primer for colony PCR and sequencing of *mmpL6*-replaced allele |
| mab-mmpl6-seqRv | cggggagtccattgggaaac | Reverse primer for colony PCR and sequencing of *mmpL6*-replaced allele |
| mab-mmpl7-seqFw | taacaagtacgccgctacctc | Forward primer for colony PCR and sequencing of *mmpL7*-replaced allele |
| mab-mmpl7-seqRv | acggtgaacatcagcaggaaa | Reverse primer for colony PCR and sequencing of *mmpL7*-replaced allele |
| mab-uvrb-seqFw | ccaggtcaagatagtggtcgg | Forward primer for colony PCR and sequencing of *uvrB*-replaced allele |
| mab-uvrb-seqRv | cttgagatcaactctgggcg | Reverse primer for colony PCR and sequencing of *uvrB* -replaced allele |
| mab-prcA-seqFw | tttcatctcgcggctgtgta | Forward primer for colony PCR and sequencing of *prcA*-replaced allele |
| mab-prcA-seqRv | acggccacatcaatacctgg | Reverse primer for colony PCR and sequencing of *prcA* -replaced allele |
| mab-recD-seqFw | gctcagatgtaaggggtcgg | Forward primer for colony PCR and sequencing of *recD*-replaced allele |
| mab-recD-seqRv | atcggttgtgcgagctagatt | Reverse primer for colony PCR and sequencing of *recD* -replaced allele |
| mab-gyrA-seqFw | cgcatcgaaccggtagacat | Forward primer for colony PCR to amplify the *gyrA* gene and for subsequent sequencing analysis. |
| mab-gyrA-seqRv | ggccgtcgtagttcggaat | Reverse primer for colony PCR to amplify the *gyrA* gene and for subsequent sequencing analysis. |
